# Follicular Helper CD4^+^ T Cells, Follicular Regulatory CD4^+^ T Cells, and Inducible Costimulator and Their Roles in Multiple Sclerosis and Experimental Autoimmune Encephalomyelitis

**DOI:** 10.1155/2021/2058964

**Published:** 2021-09-12

**Authors:** Xue Zhang, Ruli Ge, Hongliang Chen, Maxwell Ahiafor, Bin Liu, Jinbo Chen, Xueli Fan

**Affiliations:** ^1^Department of Neurology, Binzhou Medical University Hospital, Binzhou, 256603 Shandong, China; ^2^School of International Studies, Binzhou Medical University, Yantai, 264003 Shandong, China; ^3^Institute for Metabolic & Neuropsychiatric Disorders, Binzhou Medical University Hospital, Binzhou, 256603 Shandong, China

## Abstract

Follicular helper CD4^+^ T (TFH) cells are a specialized subset of effector T cells that play a central role in orchestrating adaptive immunity. TFH cells mainly promote germinal center (GC) formation, provide help to B cells for immunoglobulin affinity maturation and class-switch recombination of B cells, and facilitate production of long-lived plasma cells and memory B cells. TFH cells express the nuclear transcriptional repressor B cell lymphoma 6 (Bcl-6), the chemokine (C-X-C motif) receptor 5 (CXCR5), the CD28 family members programmed cell death protein-1 (PD-1) and inducible costimulator (ICOS) and are also responsible for the secretion of interleukin-21 (IL-21) and IL-4. Follicular regulatory CD4+ T (TFR) cells, as a regulatory counterpart of TFH cells, participate in the regulation of GC reactions. TFR cells not only express markers of TFH cells but also express markers of regulatory T (Treg) cells containing FOXP3, glucocorticoid-induced tumor necrosis factor receptor (GITR), cytotoxic T lymphocyte antigen 4 (CTLA-4), and IL-10, hence owing to the dual characteristic of TFH cells and Treg cells. ICOS, expressed on activated CD4^+^ effector T cells, participates in T cell activation, differentiation, and effector process. The expression of ICOS is highest on TFH and TFR cells, indicating it as a key regulator of humoral immunity. Multiple sclerosis (MS) is a severe autoimmune disease that affects the central nervous system and results in disability, mediated by autoreactive T cells with evolving evidence of a remarkable contribution from humoral responses. This review summarizes recent advances regarding TFH cells, TFR cells, and ICOS, as well as their functional characteristics in relation to MS.

## 1. Follicular Helper CD4^+^ T Cells

**Figure 1:**
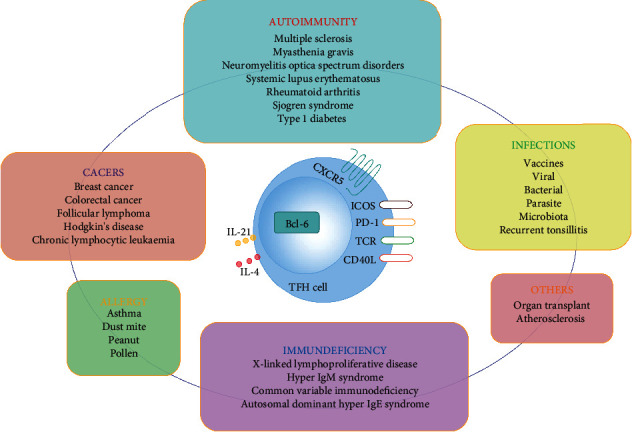
Relationships of TFH cells to diseases. TFH cells have been associated with various diseases, such as autoimmunity, cancers, infections, allergy, and immunodeficiency, with the TFH cells contributing either protective or pathogenic roles in different contexts.

Follicular helper CD4^+^ T (TFH) cells, recognized as a distinct lineage of helper CD4^+^ T cells in tonsils of humans more than a decade ago, are primarily located in germinal center (GC) and provide help for B cells to promote immunoglobulin affinity maturation, class-switch recombination, and production of memory B cells and long-lived plasma cells [1]. TFH cells specially produce high levels of interleukin-21 (IL-21) and IL-4 and self-specific transcription factor the nuclear transcriptional repressor B cell lymphoma 6 (Bcl-6) and express a unique combination of surface molecules, which include chemokine (C-X-C motif) receptor 5 (CXCR5), programmed cell death protein-1 (PD-1), and the costimulatory molecule inducible costimulator (ICOS) [2]. A great deal of recent researches have been conducted to examine the exact process of TFH cell differentiation, the cellular biology of TFH cell-mediated selection of GC B cells, and the crucial roles of TFH cells in health and disease, especially in chronic autoimmune diseases (such as multiple sclerosis and systemic lupus erythematosus), primary and acquired immunodeficiencies (for example, X-linked lymphoproliferative disease and autosomal dominant hyper IgE syndrome), infections (for instance, viral infection and bacterial infection), allergy (such as asthma and pollen allergy), cancers (for example, breast cancer and follicular lymphoma), and so on [3–8] (Figure 1).

### 1.1. Canonical TFH Cell Differentiation Pathway

Upon different stimulation, naive CD4^+^ T cell differentiation into various subsets of T helper (Th) cells relies on distinct cytokines and transcription factors (TFs) [9]. Th cells are comprised of Th1, Th2, Th17, Th22, TFH, and regulatory T (Treg) cells [10]. The differentiation of TFH cells from naive CD4^+^ T cells is a multifactorial and multistep process. In the T cell zone of secondary lymphoid tissues (SLOs), after engagement of peptide/MHC II on dendritic cells (DC) with TCR on naive CD4^+^ T cells, these T cells prime initially acquired TFH cell characteristic, including the induction of Bcl-6 expression, upregulating CXCR5 expression and downregulating chemokine (C-C motif) receptor (CCR) 7 expression [11]. Bcl-6, accompanied by other TFs (c-Maf, IRF4, and so on), induces the expression of CXCR5, ICOS, and PD-1 [12]. Simultaneously, IL-21 secreted by these naive CD4^+^ T cells, coupled with IL-6 and IL-27 produced by DC, enhances Bcl-6 and c-Maf expression in naive CD4^+^ T cells [13]. Then, these naive CD4^+^ T cells become pre-TFH cells and migrate toward the T cell-B cell border, the location of the second stage of TFH cell differentiation [14]. There, pre-TFH cells initially interact with cognate B cells to induce a high expression of Bcl-6 and CXCR5 [15]. ICOS on pre-TFH cells combines with ICOSL on B cells, thus inducing the directional migration of pre-TFH cells [2]. This process also requires the adapter signaling lymphocytic activation molecule- (SLAM-) associated protein (SAP) and SLAM family receptors Ly108 and CD84 [16, 17]. Pre-TFH and B cells together form motile conjugates which keep steady contact for minutes to hours [1]. This ensures stable localization of the cells in follicles and maintains mature TFH cell differentiation [18]. Pre-TFH cells promote B cells either differentiating into short-lived extrafollicular plasmablasts or migrating into follicles [19]. The third stage of TFH cell differentiation happens at GC, which is a particular structure comprised of GC TFH cells, GC B cells, follicular dendritic cells, macrophages, and stroma [20]. In GC, pre-TFH cells differentiate into final TFH cells also named GC TFH cells, which are CXCR5^hi^PD-1^hi^Bcl-6^hi^Maf^hi^SAP^hi^ and secrete C-X-C motif chemokine 13 (CXCL13), IL-21, and IL-4 [7]. Adhesion molecules are central to GC TFH cells, because they can regulate their localization and their interaction with CC B cells [7]. GC TFH cells not only regulate the selection of high-affinity GC B cells but also regulate the development of long-term humoral immunity [3]. It is shown that even TFH cells in the GC are not terminally differentiated [21]. They are able to shuttle between the GC and the follicle and can even eventually enter the circulation [21].

### 1.2. Different Subsets of TFH Cells

#### 1.2.1. Circulating TFH Cells

Circulating TFH (cTFH) cells, a group of CXCR5^+^CD4^+^ T cells with “TFH-like” characteristics, have been identified in peripheral blood of humans and mice [1]. cTFH cells express lower ICOS and PD-1 but do not express Bcl-6 compared with GC TFH cells [22]. Newly generated cTFH cells express low amounts of CCR7, which have an effector memory phenotype. After 1-2 weeks in the absence of antigen stimulation, antigen-specific effector memory cTFH cells can gradually transform into central memory cTFH cells with the CXCR5^+^CCR7^hi^PD-1^−^ICOS^−^ phenotype [23]. In health, approximately 80% of cTFH is the central memory phenotype, showing the low degree of active TFH cell differentiation which exists in a homeostatic state [24].

#### 1.2.2. Memory TFH Cells

Nearly 20% of human central memory CD4^+^ T cells are CXCR5^+^, indicating that memory TFH cells occupy an essential part of human T cell memory [7]. TFH cells become memory TFH cells and downregulate Bcl-6 after migrating from GC [25]. Memory TFH cells, majorly residing in the spleen, lymph node (LN), and bone marrow, exhibit a central memory phenotype and have the capacity to recirculate in blood [26]. Resting memory TFH cells express low amounts of PD-1. Upon reactivation, memory TFH cells preferentially become GC TFH cells and TFH cells [27].

#### 1.2.3. TFH1, TFH2, and TFH17 Cells

TFH cells are composed of three subsets according to the expression of chemokine receptors: CXCR3^+^CCR6^−^ TFH (TFH1) cells, CXCR3^−^CCR6^−^ TFH (TFH2) cells, and CXCR3^−^CCR6^+^ TFH (TFH17) cells [28]. The three subsets share identical signature transcription factors and cytokines of their equivalent Th cell subsets: T-bet and IFN-*γ* for Th1 and TFH1 cells; GATA3, IL-4, IL-5, and IL-13 for Th2 and TFH2 cells; and ROR*γ*t, IL-17, and IL-22 for Th17 and TFH17 cells [28]. TFH2 and TFH17 efficiently induce naive B cells to produce immunoglobulins through IL-21 [28]. TFH2 promotes the production of IgG and IgE, while TFH17 induces the production of IgG and IgA [15]. In contrast, TFH1 cells lack the capacity to help B cells [28]. Recently, a CXCR3^+^CCR6^+^ TFH cell subpopulation named TFH17.1 cells was found, which produced both IFN-*γ* and IL-17 [29].

#### 1.2.4. NKTFH Cells

NKTFH cells, recently recognized in GC, express Bcl-6, CXCR5, and PD-1 and promote B cell responses [30]. They have potential to induce memory B cell responses to T-dependent antigens [31]. NKTFH cells can provide help to promote the B cell differentiation program with features of GC formation, extrafollicular plasmablasts, affinity maturation, and IgG antibody response [31]. However, NKTFH cells do not induce the production of long-lived plasma cells or memory B cells [31].

#### 1.2.5. *γδ*TFH Cells

Human *γδ*TFH cells, the same as conventional TFH cells, express CXCR5 and are located in follicles and GCs [22]. *γδ*T cells are activated after they recognize nonpeptidic phosphoantigens which are derived from microbial metabolites; they subsequently differentiate into *γδ*TFH cells with requirement of exogenous IL-21 [32].

## 2. Follicular Regulatory CD4^+^ T Cells

Follicular regulatory CD4+ T (TFR) cells, a regulatory counterpart for TFH cells, are a subset of regulatory T (Treg) cells which have been found in the spleen, lymph nodes (LN), or other lymphoid tissues such as Peyer's patches and also in blood [33–37]. TFR cells not only share numerous TFH-related molecules including CXCR5, PD-1, Bcl-6, and ICOS but also express large amounts of Treg-related factors, such as glucocorticoid-induced tumor necrosis factor receptor (GITR), cytotoxic T-lymphocyte antigen 4 (CTLA-4), IL-10, CD25, and FOXP3, so they are different from both Treg and TFH cells [38, 39]. TFR cells lack expression of CD40L, IL-4, and IL-21 [40]. On the one hand, TFR cells control excessive GC responses through acting on TFH and GC B cells; on the other hand, their suppression of B cells occurs at diverse stages of B cell differentiation, from activation to class-switched B cells and plasma cells [41]. TFR cells not only secrete immunosuppressive cytokines IL-10 and TGF-*β* but also suppress TFH cells through CTLA-4 and control GC B cells by suppression of CD28 ligands [42]. In addition, there are cTFR (CXCR5^+^FOXP3^+^ T) cells in the peripheral blood, which own a reduced capacity to regulate humoral immunity in contrast with tissue-resident TFR cells and circulating Treg cells [43]. In conclusion, TFR cells lead to the selection of the highest affinity antigen-specific antibody and memory B cells [41].

### 2.1. TFR Cell Differentiation Pathway

It was initially proposed that TFR cells are primarily derived from thymic Treg cells [37]. In addition, TFR cells can also derive from naive Th cells in a PD-L1-dependent manner using certain adjuvants [44]. TFR cells appear to experience a multistage Bcl-6-dependent differentiation process similar to TFH cells [45]. First, the differentiation of TFR cells is initiated after interaction with activated DC in the T cell zone [41]. After that, a part of TFR cells leaves the LN, enters the peripheral circulation, and becomes a memory-like TFR cell in blood [41]. The circulating TFR cells retain a reduced suppressive function and lower expression of ICOS contrasted with TFR cells in LN [43]. Alternatively, another part of TFR cells migrates toward the T-B border and enters follicles to interact with B cells and then becomes intermediate TFR cells. The final maturation step is in the GC where TFR cells acquire a phenotype of effector TFR cells upon interaction with B cells and TFH cells [41]. Effector TFR cells which have lower expression of CXCR5, ICOS, and PD-1 than GC TFH cells suppress TFH and GC B cells and GC formation [33]. Like TFH cells, TFR cell differentiation requires TCR stimulation and costimulatory molecules CD28 and ICOS which are important for TFR differentiation in both the T cell zone and follicle/GC [33]. IL21 has a negative role in TFR cells while it promotes TFH cell differentiation [46].

Although TFH and TFR cells are characterized by their anatomical location in secondary lymphoid tissues, an increasing number of experiments have shown putative circulating counterparts of these cells in peripheral blood especially for human samples. It is limiting to have access to secondary lymphoid tissues. A recent study showed that circulating TFR cells were increased in Sjögren syndrome (SS), a systemic autoimmune disease characterized by ongoing GC reactions [43]. In addition, there was a substantial increase in the TFR/TFH ratio in SS patients, which was associated with serum autoantibodies of SS patients [43]. Another study indicated that the circulating TFR/TFH ratio and activated TFH cells were associated with pathological lymphocytic infiltration in the SS target organ minor salivary gland (MSG) and disease activity, respectively, in primary SS [47]. On account of the regulatory role of TFR cells, the predictive value of TFR cells and TFR/TFH ratio in SS patients may appear controversial [48]. Fonseca et al. showed that human blood TFR cells remain immature, generated prior to T-B interactions, lack full B cell-suppressive capacity, and have a naïve-like phenotype, although they correlated with humoral responses [43]. Nevertheless, the frequency of blood TFR cell and the TFR/TFH ratio were reduced in systemic lupus erythematosus (SLE) and rheumatoid arthritis (RA) patients [49, 50]. The TFR/TFH ratio and the frequency of TFR cells were negatively correlated with disease activity and serum anti-dsDNA antibody level in SLE [49]. Moreover, the TFR/TFH ratio was negatively correlated with values of ESR, RF, CRP, and DAS28 scores while it was positively correlated with serum TGF-*β* level [50]. In conclusion, the TFH, TFR, and TFR/TFH ratio may be responsible for the immunopathogenesis of diseases.

## 3. Inducible Costimulator (ICOS)

**Figure 2:**
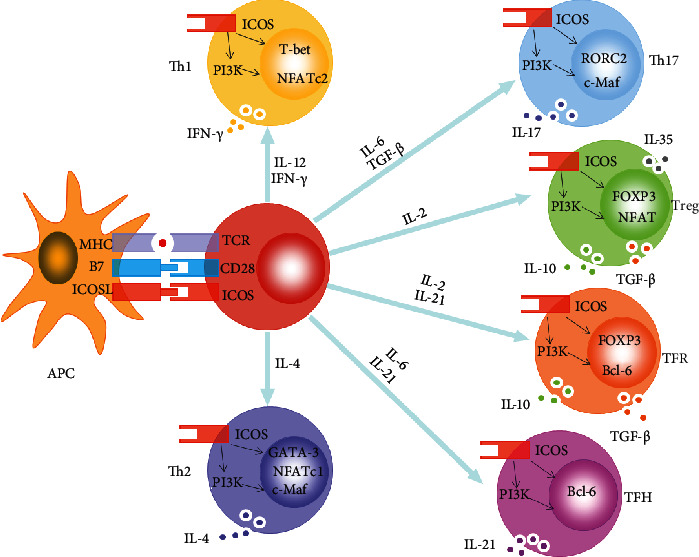
ICOS induces the differentiation of CD4^+^ T cell subsets. For Th1 cells, ICOS ligation upregulates transcription factors NFATc2 and T-bet and promotes IFN-*γ* production. For Th2 cells, ICOS engagement upregulates transcription factors NFATc1 and c-Maf and promotes GATA-3 transcription and the production of IL-4. Moreover, binding of ICOS to ICOSL promotes FOXP3 transcription, favoring NFAT binding to FOXP3 in Treg cells, and promotes IL-10, IL-35, and TGF-*β* secretion. In addition, ICOS-ICOSL interaction induces RORC2 and c-Maf expression and promotes IL-17 production in Th17 cells, whereas binding of ICOS to ICOSL induces Bcl-6 expression in TFH cells, leading to increased secretion of IL-21. What is more, ICOS ligation upregulates transcription factors FOXP3 and Bcl-6 and promotes IL-10 and TGF-*β* production in TFR cells.

According to the two-signal model for T cell activation, T cells first require an antigen-specific signal and then a costimulatory signal in order to induce an optimal adaptive immunity [51] . The costimulatory signals are essential for T cell proliferation, lymphokine secretion, and effector function [52]. ICOS, as a member of the CD28 family of costimulatory molecules, is expressed at low levels on resting naive T cells and is rapidly induced following TCR ligation and CD28 costimulation [53]. Then, ICOS has a high expression on activated T cells and delivers a positive costimulatory signal [54]. ICOS signal is initiated upon ligation with the ICOS ligand (ICOSL) which is expressed on antigen-presenting cells, including B cells, DCs, macrophages, certain endothelial cells, and lung epithelium [55]. ICOS ligation induces phosphatidylinositol 3-kinase (PI3K) signaling which contributes to the production of membrane-bound phosphatidylinositol 3,4,5-trisphosphate (PIP3), followed by the activation of Akt—a kinase that promotes cellular proliferation and survival [56, 57]. So, the engagement of ICOS promotes cell differentiation, proliferation, and survival [58]. ICOS can induce the differentiation of Th1, Th2, Th17, TFH, Treg, and TFR cells depending on different contexts of the inflammatory circumstances, because ICOS coinduces the production of IFN-*γ*, TNF-*α*, IL-4, IL-5, IL-6, IL-10, IL-21, and so on [53] (Figure 2). Activation of ICOS upregulates transcription factors NFATc2 and T-bet in Th1 cells and promotes the secretion of IFN-*γ*, whereas in Th2 cells, binding of ICOS to ICOSL upregulates transcription factors NFATc1 and c-Maf and promotes GATA-3 transcription and IL-4 secretion [53, 59] (Figure 2). ICOS-ICOSL interaction promotes FOXP3 transcription, favors NFAT binding to FOXP3, and promotes the production of IL-10 in Treg cells [60] (Figure 2). Moreover, ICOS stimulation induces RORC2 and c-Maf expression in Th17 cells and Bcl-6 expression in TFH cells, leading to increased secretion of IL-17 and IL-21, respectively [56, 61]. What is more, ICOS ligation upregulates transcription factors FOXP3 and Bcl-6 and promotes IL-10 and TGF-*β* production in TFR cells [62] (Figure 2).

### 3.1. ICOS and TFH Cells

**Figure 3:**
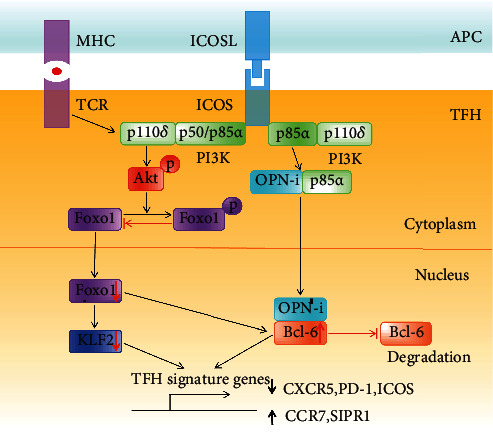
Signaling pathway for ICOS costimulation in TFH cell differentiation. For TFH cell differentiation, ICOS-mediated recruitment of PI3K has a dual role in transcriptional regulation of genes. On the one hand, the activation of PI3K causes activation of Akt and subsequently inhibits the transcription factor Foxo1 function through phosphorylation-mediated cytosolic retention. The inhibition of Foxo1 upregulates Bcl-6 and downregulates KLF2, which both are crucial transcriptional regulators controlling the expression of TFH signature genes. On the other hand, the regulatory subunit p85*α* of PI3K can bind with the intracellular form of osteopontin (OPNi), whereafter OPNi migrates into the nucleus and binds to Bcl-6 which can protect Bcl-6 from ubiquitin-dependent degradation and regulate the expression of TFH signature genes.

ICOS was first found on the cell surface of GC T cells, indicating its relevant role in T cell-B cell interactions [56]. Signals through ICOS-ICOSL are crucial for each stage of TFH cell differentiation and function [63]. For the differentiation and maintenance of TFH cells, ICOS can recruit PI3K which has a dual role in transcriptional regulation of genes [64] (Figure 3). On the one hand, activation of Akt through the p110*δ* catalytic subunit of PI3K phosphorylates the transcription factor Foxo1, which is accordingly retained in the cytoplasm [65] (Figure 3). This timely inhibition of Foxo1 upregulates Bcl-6 and downregulates KLF2, which both are vital transcriptional regulators controlling the expression of TFH signature genes involved in TFH migration and function [66]. On the other hand, the PI3K regulatory subunit p85*α* forms complexes with the intracellular forms of osteopontin (OPNi) which migrates into the nucleus and binds to Bcl-6 in order to protect Bcl-6 from ubiquitin-dependent degradation [67] (Figure 3). Besides, ICOS signaling can also affect IL-21 production via c-Maf, thereby regulating TFH cell differentiation [63].

ICOS-mediated signals are not only necessary for TFH differentiation but also required for TFH migration and T-B collaboration [64]. At the T-B border, the ligation of ICOS on pre-TFH cells and ICOSL on bystander B cells that do not present cognate antigens promotes T cell motility allowing cognate T-B conjugation and GC enter [64]. This process depends on the ICOS-PI3K signaling and presumably activation of small GTPases directly and/or indirectly by ICOS [68]. In the context of high-affinity interactions between the TCR and peptide-MHC complexes, ICOS ligation allows focused delivery of T cell “help” to cognate GC B cells within the GC. The ICOS-PI3K-mTOR signaling has been shown to facilitate IL-4 production from existing mRNA by inducing polysome formation [69]. Meanwhile, ICOS-mediated calcium flux promotes CD40L externalization providing CD40 costimulation to the differentiation of GC B cells [70]. In turn, CD40 signaling elevates ICOSL expression level. This feedforward cross-talk promotes selection of high-affinity GC B cell clones [64].

ICOS is regarded as a marker of TFH cells in human [71]. Increased frequency of ICOS^+^ or ICOS^high^ cTFH cells standing for an “activated” phenotype has been found in patients with autoimmunity such as RA [72], SS [73], myasthenia gravis (MG) [74], SLE [75], autoimmune thyroid diseases [76], and all autoimmune pathologies partially caused by autoantibodies [1]. Interestingly, increased frequency of TFH cells was positively correlated with tissue damage, high titers of autoantibodies, IL-21 levels, and frequencies of circulating GC B cells and plasma cells in these pathologies [4]. Blockade of ICOS-ICOSL signaling can ameliorate disease through disrupting TFH cell responses in SLE, MG, collagen-induced arthritis, allergic asthma, and pemphigus vulgaris [77–79]. Thus, TFH and ICOS, involved in pathogenic mechanisms, may be potential therapeutic targets in autoimmune diseases.

### 3.2. ICOS and TFR Cells

ICOS is essential for TFR differentiation and function [64]. First, ICOS-ICOSL interaction induces FOXP3 transcription, favoring the transcription factor NFAT combining with FOXP3 and upregulating FOXP3 downstream regulatory genes [60]. Second, elevation of PI3K- mammalian target of rapamycin (mTOR) after ICOS ligation induces Treg conversion to the TFR lineage in Roquin-deficient mice [80]. In addition, the ICOS-calcium pathway plays a crucial role in the development of TFR cells since the calcium-dependent transcription factor NFAT2 is essential to upregulate CXCR5 in TFR cells [81]. What is more, impaired calcium signaling caused by Stim1 and Stim 2 deletion resulted in reduced TFR cell differentiation and spontaneous autoantibody production [82]. Thus, these data indicate that ICOS signaling contribute to TFR generation.

## 4. TFH Cells, TFR Cells, and ICOS in Multiple Sclerosis and Experimental Autoimmune Encephalomyelitis

Multiple sclerosis (MS), one of the most common chronic immune-mediated diseases of the central nervous system (CNS), is affecting more than 2.5 million people worldwide [83]. MS is characterized by inflammation of CNS and demyelination of neurons and causes collateral damage in the neighboring CNS system [84]. There are three crucial disease phenotypes of MS which are traditionally recognized: relapsing-remitting MS (RRMS), secondary progressive MS (SPMS), and primary progressive (PPMS) [85]. MS majorly affects individuals in their early adult life, so it has an enormous impact functionally and financially on the quality of life [86]. Experimental autoimmune encephalomyelitis (EAE), one of the most widely used MS animal models, is used to explore the pathogenesis of MS.

Although the exact etiology remains unknown, current researches have suggested that genetic and environmental factors, including low vitamin D levels, cigarette smoking, and obesity, have causal roles in MS [86]. Previously, autoreactive T cells are regarded as the central drivers of MS pathogenesis and progression [87]. Nevertheless, recent studies have shown that the interplay between B and T cells is an essential player in immune responses involved in MS [88]. According to the researches, B cells not only present antigen to T cells and promote autoproliferation of brain-homing T cells but also produce soluble toxic factor and proinflammatory cytokines and chemokines in MS [89]. In addition, there are intrathecal antibodies and B cell-rich aggregates in the meninges surrounding the brain and spinal cord, which are essential contributors of CNS compartmentalized inflammation [89]. Moreover, CD19/20 monoclonals depleting B cells are effective for MS patients [85].

Thus, understanding the role of TFH cells, TFR cells, and surface molecule ICOS which were involved in the interaction between B and T cells is important in looking for new therapeutic strategies in MS and EAE.

### 4.1. TFH Cells, TFR Cells, and ICOS in Multiple Sclerosis

Guo et al. found that the proportion of circulating TFH cells (CD3^+^CD4^+^CXCR5^+^PD-1^+^) was higher in relapsing RRMS patients than in remitting RRMS and healthy controls (HCs) [90]. The level of circulating TFH cells was positively correlated with the level of serum IL21 and B cells [90]. Another study showed that the numbers of circulating ICOS^+^, CCR7^+^, and CCR7^+^ICOS^+^ memory TFH cells, as well as plasma IL21 level, were higher in MS patients than in HCs [91]. In addition, the frequency of circulating TFH1 cells is decreased in MS patients compared to HCs, and the frequency of circulating TFH17 cells was reduced in PPMS [92]. A proinflammatory bias presented higher frequencies of circulating TFH17.1 cells and lower frequencies of TFH2 cells, which was found in RRMS patients compared to HCs [93]. In addition, IL21 and IL21R are involved in the immunopathogenesis of MS [94]. An experiment showed that the levels of serum IL21 and IL-21 mRNA were significantly increased in MS patients compared with HCs and were positively correlated with EDSS scores and progression index of MS patients [95]. Moreover, the increased frequencies of IL21-producing TFH cells were positively related to MS severity and progression [95]. Another study reported that the frequencies of circulating TFR cells in MS patients were significantly lower than HCs [96]. Furthermore, TFR cells in MS patients owned an obviously reduced suppressive function compared with HCs, indicating prominent TFR cell impairment in MS [96]. Similarly, another study also indicated lower TFR frequencies and higher TFH/TFR ratio in RRMS patients at the clinical onset compared with HCs [97]. The TFH/TFR ratio, as a matter of fact, is positively associated with abnormal IgG synthesis in serum and CSF in RRMS patients, indicating that autoreactive B cell clones, derived from deregulated peripheral GC reaction, may colonize the CNS [97]. TFH cells can migrate to the CNS in MS patients [98]. There is evidence that the levels of CXCL13 and the chemokine ligand for CXCR5 on TFH cells and T cells were increased in the CSF of MS patients, suggesting that TFH cells migrate and stay in the CSF because of elevated CXCL13 expression [98]. A recent study showed a cluster-independent increase in TFH cells potentially driving the MS-specific B cell expansion in the CSF of MS through cell set enrichment analysis [99]. They found an obviously increased percentage of TFH, PD-1^+^ TFH, and PD-1^+^ICOS^+^ TFH cells in the CSF of MS patients by flow cytometry, while the proportion of PD-1^+^ TFH cells had a positive relation to the percentage of CSF plasma cells and disease progression [99]. Another study indicated that the increased frequency of memory TFH cells in CD4^+^ T cells in the CSF of RRMS has a significant correlation with the percentage of antibody-secreting B cells [100].

A recent study showed that abatacept, a CTLA-4-Ig fusion protein inhibiting T cell activation and function, reduced the relative frequencies of circulating CD45RO^+^ TFH cells expressing the activation markers CD38 and ICOS in patients with RRMS [101]. Dimethyl fumarate (DMF), an oral fumaric acid ester, is a disease-modifying therapy for RRMS. MS patients had reduced proportions of TFH cells and antigen-experienced B cells, accompanied by increased TFR cells in DMF-treated MS patients compared to untreated patients [102]. Another study showed that DMF treatment resulted in a progressive increase in cTFH2 cells, together with a decrease in cTFH1 and the pathogenic cTFH17.1 cells, indicating a possibly pathogenic cTFH proinflammatory profile of RRMS [93].

### 4.2. TFH Cells, TFR Cells, and ICOS in Experimental Autoimmune Encephalomyelitis

It is reported that the frequency of PD-1^+^ and ICOS^+^ TFH cells in the spleen and draining lymph nodes increased before the manifestation of clinical symptoms, attained a maximum during the peak stage, and declined at the remission stage of EAE [90]. The percentage of these TFH cells elevated slightly in the chronic phase [90]. What is more, the protein levels of Bcl-6, CXCR5, and IL21, together with the serum IL-21 level, presented the similar changes as those of TFH cells [90]. Both the frequencies of TFH cells and the serum level of IL21 had positive correlations with the disease scores of EAE [90]. In Th17-induced EAE, there are a massive number of PD-1^+^ TFH cells in the CNS tissue which was correlated with the number of infiltrating B cells at the peak of disease [103]. TFH cells induce B cell infiltration into the CNS and locally drive B cell response in the CNS of EAE [99]. The proportion of PD-1^+^ TFH cells in the spinal cord and brain reached a maximum at the peak phase of EAE and had a positive correlation with the disease score [90]. A number of ICOS^+^ TFH and PD-1^+^ TFH cells were also found within the ectopic lymphoid structures of spinal cords in EAE. Moreover, TFH cells had a great potential in boosting anti-MOG antibody production of B cells via IL-21 and CD40 ligand (CD40L) and the synergy effect of the STAT3 and noncanonical NF-*κ*B signaling pathway inside B cells [90]. Besides, adoptive transfer of TFH cells could enhance the disease severity and delay disease remission [90]. Interestingly, mice with a FOXP3-specific deletion of Blimp1 develop severe EAE and increased TFH-B-Ab response, while having increased frequency of TFR cells [104]. Most TFH cells were TFH17cells and expressed more IL-17A and GM-CSF, as well as CXCL13 in *Prdm1^fl/fl^FOXP3^Cre^* EAE mice [104]. In contrary to WT TFR cells, Blimp1-deleted TFR cells were more encephalitogenic, reflecting dysregulated Ab response and EAE progression [104].

A previous study showed that blockade of the ICOS/ICOSL pathway during the efferent immune response (9–20 days after immunization) alleviated the disease, but ICOS blockade during the antigen priming stage (1–10 days after immunization) aggravated the disease in proteolipid protein- (PLP-) induced EAE [105]. For the former, ICOS blockage inhibited the activation and proliferation of the encephalitogenic ICOS^+^ T cells and restrained the following recruitment of nonantigen-specific leukocytes in the efferent immune stage [105]. In the latter however, blockade of the ICOS/ICOSL pathway in antigen priming response resulted in an increase in the ratio of Th1 : Th2 cells and amplified the activation of monocytes or macrophages and microglia [105]. In another study, the researchers used an antagonistic antibody against CXCL13, the chemokine ligand for CXCR5 on TFH cells to treat Th17-EAE in order to block TFH trafficking, and found that it can significantly reduce TH17-EAE disease [106]. A recent study showed that laquinimod suppressed TFH (PD-1^+^CXCR5^+^BCL-6^+^ TFH) and B cell aggregates and reduced disease progression in EAE [107].

In summary, TFH cells, TFR cells, and ICOS are all involved in the pathological process of MS and EAE and are regarded as markers of disease severity, progression, and prognosis. Hence, TFH cells, TFR cells, and ICOS may be beneficial therapeutic targets of the disease. Monitoring TFH cells and TFR cells in the treatment process of MS is supposed to become a biological marker of drug efficacy.

## 5. Conclusion

TFH cells, TFR cells, and ICOS play significant roles in the pathogenesis of various diseases. TFH cells, TFR cells, and ICOS might be the potentially key targets for novel therapeutic selections in diseases. Further studies are still needed to better understand the precise roles of them in diseases, which will open a new avenue to explore the mechanisms of the pathogenic process in diseases.
